# BRG1: Promoter or Suppressor of Cancer? The Outcome of BRG1’s Interaction with Specific Cellular Pathways

**DOI:** 10.3390/ijms24032869

**Published:** 2023-02-02

**Authors:** Aaron Shaykevich, Isaac Silverman, Gargi Bandyopadhyaya, Radhashree Maitra

**Affiliations:** Department of Biology, Yeshiva University, New York, NY 10033, USA

**Keywords:** BRG1, SMARCA4, cancer, autophagy, apoptosis, PRMT5, KRAS, ERK

## Abstract

BRG1 is one of two catalytic subunits of the SWI/SNF ATP-dependent chromatin-remodeling complex. In cancer, it has been hypothesized that BRG1 acts as a tumor suppressor. Further study has shown that, under certain circumstances, BRG1 acts as an oncogene. Targeted knockout of BRG1 has proven successful in most cancers in suppressing tumor growth and proliferation. Furthermore, BRG1 effects cancer proliferation in oncogenic *KRAS* mutated cancers, with varying directionality. Thus, dissecting BRG1’s interaction with various cellular pathways can highlight possible intermediates that can facilitate the design of different treatment methods, including BRG1 inhibition. Autophagy and apoptosis are two important cellular responses to stress. BRG1 plays a direct role in autophagy and apoptosis and likely promotes autophagy and suppresses apoptosis, supporting unfettered cancer growth. PRMT5 inhibits transcription by interacting with ATP-dependent chromatin remodeling complexes, such as SWI/SNF. When PRMT5 associates with the SWI/SNF complex, including BRG1, it represses tumor suppressor genes. The Ras/Raf/MAPK/ERK1/2 pathway in cancers is a signal transduction pathway involved in the transcription of genes related to cancer survival. BRG1 has been shown to effect KRAS-driven cancer growth. BRG1 associates with several proteins within the signal transduction pathway. In this review, we analyze BRG1 as a promising target for cancer inhibition and possible synergy with other cancer treatments.

## 1. Introduction

Brahma-related gene-1 (BRG1) is utilized as a catalytic subunit by a variety of enzymatic complexes which modify chromatin structure [1]. *SMARCA4* (also referred to in this article as BRG1) is the gene encoding for BRG1 expression. Chromatin units restructured by such complexes containing BRG1 have been observed to both trigger and hinder gene expression. Additionally, BRG1 has proven to play a role in complexes that signal for gene silencing of certain promoter regions, thus repressing transcriptional function [2]. ATP hydrolysis provides the necessary energy for such chromatin remodeling complexes which contribute to the reformation of the nucleosome [3].

BRG1 is one of two catalytic subunits used by the SWI/SNF complex, the other catalytic subunit being hBRM [4]. The SWI/SNF complex is one of the major ATP hydrolysis-dependent chromatin remodeling complexes [5]. The complex has been observed to serve as an enzyme mechanism for gene expression regulation. It has been demonstrated that SWI/SNF is involved in nucleosomal structure alteration, which exposes binding sites for transcription factors. Further studies have revealed that SWI/SNF participates in mechanisms that direct it toward targeted promoters which have the potential to either activate or repress transcription [6]. Notably, experiments have shown that, when separated from the SWI/SNF complex, BRG1 is capable of functioning alongside as few as one other SWI/SNF subunit to accomplish transcription activation as well as chromatin modification [7].

The BRG1 subunit has been suspected to be capable of suppressing tumor formation. BRG1 has been found to function with BRM and interact with the protein product of the Retinoblastoma tumor suppressor gene product to repress the E2F transcription factor function [8]. Furthermore, a homozygous deletion of the carboxylic acid terminal region of BRG1 has been observed in prostate and lung carcinoma cell lines. Biallelic activations of BRG1 have also been discovered in several cancer cell lines including prostate, lung, breast, and pancreatic [9]. Tumor suppression ability has also been discovered with other subunits within the SWI/SNF complex, adding further legitimacy that the complex is connected with tumor suppression and regulation of cell growth [10].

Although BRG1 has been found to act as a tumor suppressor, more recent research calls attention to its role as a tumor promoter. Many studies have proven that the expression of BRG1 is upregulated in cancers compared with healthy tissue [11,12,13]. BRG1’s role as a tumor suppressor is, therefore, not its only role in cancer, and BRG1 has been discovered to be an oncogene [14,15]. In fact, BRG1 overexpression in cancers can be used as a prognostic indicator. With most cancers, including breast cancer, colorectal cancer (CRC), and prostate cancer, BRG1 upregulation is correlated with worse outcomes, underlining BRG1 as an oncogene [12,16,17,18,19,20]. However, patients with Non-small Cell Lung Cancer (NSCLC) who lacked BRG1 production had a worse prognosis than those with normal BRG1 expression, leading to divergent roles of BRG1 as tumor suppressor/promoter in different cancer types [21].

While BRG1 treatments have been promising, a more comprehensive understanding of BRG1’s effect on certain cancer pathways may allow for dual treatments consisting of BRG1 inhibition and upregulating/downregulating another pathway involved in cancer proliferation. Autophagy, apoptosis, PRMT5, and the RAS/RAF/ERK1/2 pathway are most significantly of interest to us. Analysis of autophagy and apoptosis with connection to BRG1 allows for an understanding of the mechanism behind BRG1’s role in cancer formation. PRMT5 is a protein with significant effects on cancer growth and is highlighted in this review to emphasize the role BRG1 plays alongside it, whether cancer growth or inhibition. The RAS/RAF/ERK1/2 pathway is a signal transduction pathway with significant importance to researchers. *KRAS*, one of the three RAS genes, for example, is a necessary protein to study due to its frequent mutations in certain cancers.

This review will attempt to demonstrate the multifaceted effects BRG1 has on cancer sustainability and vitality. Treatment via knockdown of BRG1 is a growing trend for researchers of some cancers, and a synergistic approach via dual treatment may assist in cancer inhibition.

## 2. BRG1 Acts as a Cancer Promoter via Autophagy and Apoptosis Pathways

BRG1’s potential role as an oncogene has prompted research in BRG1 drug and knockout treatments in several types of cancer. Knockdown of BRG1 in prostate cancer has been observed to have an inhibitory effect on tumor growth [19]. In CRC, the knockdown of BRG1 induces cell senescence [22] and apoptosis [20]. In triple-negative breast cancer, BRG1 knockdown has played a supporting role in tumor management in conjunction with chemotherapy treatment [23]. However, in NSCLC, knockout of BRG1 in lung cancer correlates with tumor progression, not suppression [24]. With the research of BRG1 targeted therapies becoming more promising, it is important to understand BRG1’s role in cellular autophagy and apoptosis and the pathways involved.

Macroautophagy (referred to in this article as autophagy) should be of interest to researchers due to its nature as both a cancer proliferator and a cancer inhibitor. Autophagy is predominately triggered by nutrient starvation [25,26]. It is first initiated via the ULK1 complex, including proteins ULK1, ATG13, and FIP200. mTORC1 hyperphosphorylates ATG13, preventing autophagy in cells not undergoing starvation. The ULK1 complex initiates the VPS34/Beclin 1 kinase complex, containing VPS34, Beclin 1, VPS15, and ATG14L. This complex produces phospholipid PI3P, which binds with proteins to create the autophagosome. The ATG16L1–ATG5–ATG12 conjugation machinery and LC3 conjugated systems work with many ATG’s to expand the autophagosome [27,28].

Autophagy is generally considered a mechanism of tumor growth and metastasis because it is an attempt by the cell to maintain viability. Mutations of autophagy genes are quite rare in cancer [29], which suggests that autophagy plays an important role in cancer development. Previous findings have found a mutation of the Beclin1 gene in breast, ovarian, and prostate cancer which provides evidence to support the hypothesis of autophagy functioning as a tumor suppressor [28]. However, others have disputed the explanation, since Beclin1 is adjacent to BRCA1, a tumor suppressor gene [29]. The debate over autophagy as a tumor suppressor or promoter is still unresolved. Therapies promoting and inhibiting autophagy are both being attempted.

Apoptosis is a cellular process initiating a programmed cell death, which consists of two main pathways: the extrinsic and intrinsic pathways. The intrinsic pathway is mediated by the mitochondria. This mediation can involve negative signals, such as a lack of growth factors, hormones, and cytokines, and positive signals, including an abundance of radiation, toxins, hypoxia, hyperthermia, viral infections, or free radicals. The extrinsic pathway involves transmembrane death receptors [30].

BRG1 has a prominent role in autophagy and apoptosis regulation. A comprehensive understanding of the proteins and pathways BRG1 utilizes will educate researchers on how BRG1 acts as a cancer proliferator, as well as what proteins are upregulated. This may allow for treatment methods designed to target the cell’s ability to regenerate and induce cellular death.

### 2.1. BRG1 and Autophagy

The relationship between BRG1 and autophagy is complex, with a lack of investigation into the topic. Even less information has been discovered about the direct interactions between BRG1 and autophagic proteins. However, one study of intestinal epithelial cells did share findings of a meaningful connection between BRG1 and autophagy proteins. Cells lacking proper BRG1 expression had significantly reduced autophagy. This study also found a direct correlation between the deletion of BRG1 and a decrease in key autophagy regulatory genes, mainly ATG16L1, ATG7, Ambra1, and WIPI2 [31].

ATG16L1 and ATG7 are direct components of the ATG16L1–ATG5–ATG12 conjugation system. These proteins assist in LC3 phosphatidylethanolamine conjugation and autophagosome formation [31]. Ambra1 stabilizes the VPS34/Beclin 1 complex by binding to Beclin1, assisting in the formation of autophagosomes. It is inferred that Ambra1 is necessary for autophagy, as Ambra1 deficiency is correlated with impaired autophagy [32]. WIPI2 is one of four WIPI proteins which comprise PROPPIN (β-propellers that bind polyphosphoinositides). WIPI binds to PI3P as a method of signaling autophagic molecules. WIPI also assists in the lipidation of LC3 by recruiting and binding to the ATG16L1–ATG5–ATG12 complex [33].

BRG1 has been documented to interact with p53 [22,31,34]. p53 is a regulatory protein involved with both autophagy and apoptosis. In CRC, BRG1 has been shown to bind to SIRT-1 and enhance SIRT1-mediated deacetylation of p53 at K382. The knockout of BRG1 correlates with less efficient SIRT-1 deacetylation and increased stability in p53 [22]. Another study of CRC found that BRG1 knockdown led to increased p53 expression and found evidence of a BRG1/CHD4/HDAC1 complex regulating p53 transcription and stability [34].

Retinoblastoma protein (RB) and BRG1 also interact to initiate autophagy. RB is a cancer suppressor protein, initiating cell cycle arrest at the G1 checkpoint [35]. Together with BRG1, RB suppresses E2F1 activation functions [36]. E2F1 acts as an antagonist to RB-mediated autophagy. Therefore, the BRG1/RB suppression of E2F1 initiates autophagy [37]. Furthermore, BRG1 has been found to enhance RB inhibition of E2F and cyclin A [38]. This suggests that BRG1 may cooperate further with RB for autophagy initiation.

Contrary to studies supporting BRG1 as an autophagy inducer, some studies have reported that BRG1 functions as an autophagy inhibitor. A study of the hearts of mice found that mice with double mutant BRG1 & BRM showed an increase in mitochondrial autophagy (mitophagy) [39]. This study differs in that BRM is mutated alongside BRG1. Therefore, there may be differences in autophagic flux when both SWI/SNF ATPase subunits are affected as opposed to just BRG1. A different study of renal fibrosis in vivo and in vitro found that overexpression of BRG1 inhibited autophagy and suggested this is accomplished through activation of the Wnt/β-catenin signaling pathway [40]. Further study of this pathway in varying cancer types in the context of BRG1 and autophagy is necessary for clarification of BRG1’s role in autophagy. 

**Figure 1:**
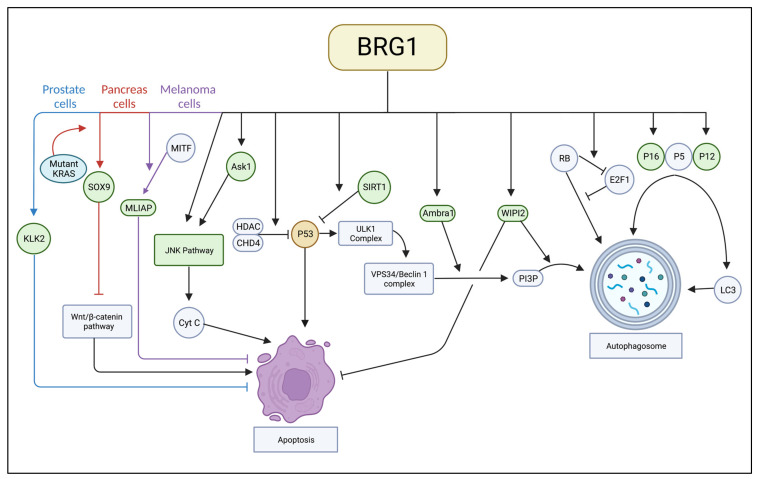
A complete image of interactions between BRG1 and apoptotic and autophagic proteins.

Most research suggests BRG1 has a role in supporting autophagy at different stages of autophagy. However, more research into BRG1’s effect on various cancer types is necessary to establish conclusive interactions and methods of treatment. The interactions BRG1 has with autophagic proteins are depicted in Figure 1.

### 2.2. BRG1 and Apoptosis

BRG1’s relationship with apoptosis has been documented with predominantly consistent results. Many studies have found that BRG1 expression is correlated with reduced apoptosis, and a BRG1 deficiency increases apoptosis [20,31,34,41,42]. However, other studies have concluded that the loss of BRG1 in CRC and lung tumors had no significant effect on apoptosis [22,43]. The focus of these studies, however, was not BRG1’s role on apoptosis, and each only performed one experiment documenting these results. Therefore, it appears most likely that BRG1 plays a role in apoptosis inhibition.

A study of BRG1 in CRC found that BRG1 suppression leads to upregulation in the JNK pathway [44]. The JNK pathway is a MAPK pathway that transduces extracellular signals. JNK is traditionally considered an apoptosis inducer through the intrinsic pathway by cytochrome c and caspase 3 activation [45]. A study of neuronal crest cells, which identified BRG1 as a transcriptional activator of PlexinA2, also found that BRG1 suppresses Ask1 and P21 [46]. Ask1 (apoptosis signal-regulating kinase 1) is a MAPKKK protein that induces apoptosis mainly through the JNK pathway [47]. The method by which BRG1 suppresses Ask1 is not complete, and it is not specified if this is a cell-specific interaction.

Several studies have noted cancer-specific BRG1 apoptosis pathways. A study of melanoma cells exposed to UV light found that BRG1 inhibited apoptosis alongside MITF to permit transcription of the melanoma inhibitor of apoptosis promoter ML-IAP [48]. Additionally, a study of prostate cancer cells with inhibited BRG1 found that several of BRG1’s target genes were decreased, including protein KLK2 [19], a known apoptosis inhibitor in prostate cancer [49]. Furthermore, a study of pancreatic ductal adenocarcinomas (PDAs) derived from intraepithelial neoplasia (panIN) in KRAS mutant cells found that BRG1 assisted in the initiation of panIN and its progression to pancreatic carcinoma [50]. BRG1 binds to the SOX9 promoter and helps initiate SOX9 transcription. SOX9 has been shown to have an effect on cancer proliferation, and SOX9 knockdown has a proapoptotic effect [51]. The inhibition of BRG1 has been observed to prevent panIN formation and prevent panIN-derived PDA through apoptosis induction. While the mechanism of BRG1’s inhibition of panIN apoptosis is not investigated further, SOX 9 may be another factor due to its inhibition of apoptosis through the Wnt/β-catenin signaling pathway.

p53 is an apoptotic protein, discussed previously regarding autophagy, regulated by BRG1. p53 is known to induce apoptosis in early-stage tumor cells and cells with defective autophagy [52]. Another autophagic protein regulated by BRG1 that is involved in apoptosis inhibition is WIPI2. In a study of hepatocellular carcinoma (HCC) cells, the deletion of WIPI2 promoted apoptosis [53]. By upregulating WIPI2, BRG1 has another pathway to inhibit apoptosis.

BRG1’s role as an apoptosis inhibitor has general and cancer-specific components. While the literature is not unanimous that BRG1 and apoptosis have a connection, treatment including BRG1 inhibition along with apoptosis induction may reap some unforeseen benefits. The interactions BRG1 has with apoptotic proteins are depicted in Figure 1. 

## 3. BRG1’s Interaction with PRMT5 in Cancer Development 

Protein arginine methyltransferases (PRMTs) participate in several cellular pathways used for cell maintenance. Numerous studies have found an upregulation of PRMTs in cancer cells [54]. In particular, histone methylation involving Protein Arginine Methyltransferase 5 (PRMT5) is thought to repress transcription and has been observed to interact with several gene repression complexes [55] including some chromatin remodeling complexes which are dependent on ATP hydrolysis [56]. Notably, PRMT5 has been shown to interact with the SWI/SNF complex, as well as BRG1 specifically [57]. Together, they can perform methylation on the N terminus tails of the H3 and H4 histones [58]. Methylation of histone tails, such as H3 has been observed to have significant effects on transcription [59]. Also, it has been experimentally demonstrated that the tumor suppressor genes suppressor of tumorigenicity 7 (ST7) and nonmetastatic 23 (NM23) are both repressed because of histone modification by PRMT5 when in association with BRG1 and hBRM [60]. Furthermore, PRMT5 has been shown to induce autophagy and inhibit apoptosis in certain cancers [61,62].

Several cellular processes are used to tightly coil DNA around nucleosomes and densely pack them together into histone packages for condensed storage. The core histones H2A, H2B, H3, and H4, form octamers each containing 145–147 base pairs of DNA [63]. Histone-modifying enzyme complexes work alongside ATP hydrolysis-dependent chromatin remodeling complexes to regulate transcriptional processes [3]. Histone modification can transpire through multiple mechanisms, including methylation, acetylation, phosphorylation, ubiquitination, and ADP-ribosylation. These processes affect the tail ends of histone proteins after the translation process and are suspected to influence chromatin structure. Lysine and arginine residues of H3 and H4 histones are primarily edited through methylation, although the enzyme mechanism is not fully understood [64]. As aforementioned, PRMT5 has been observed to function in histone methylation alongside SWI/SNF and BRG1, and it is therefore worth investigating their association. [57] Additionally, it is notable that deacetylation of histone H4 through Histone deacetylase 3 (HDAC 3) has been observed to significantly affect the silencing of apoptotic genes. [65]

BRG1 has been observed to function as both a tumor promoter and suppressor in association with specific protein complexes prior to oncogenesis. Often involved in those complexes is PRMT5 which contributes a histone methylation component. Histone methylation is a versatile cellular function which affects interaction between BRG1 component complexes and target genes which are associated with tumor regulation.

### 3.1. BRG1 Potentiates Cancer Development in Association with PRMT5 via H3 and H4 Histone Proteins

Numerous experiments have confirmed that methylation of histone 3 arginine 8 (H3R8) and histone 4 arginine 3 (H4R3) through PRMT5 interaction represses the transcriptional abilities of the genes targeted. Furthermore, it has been observed in cellular differentiation studies that methylation of histones through PRMT5 also has a role in transcriptional activation [57]. 

**Figure 2:**
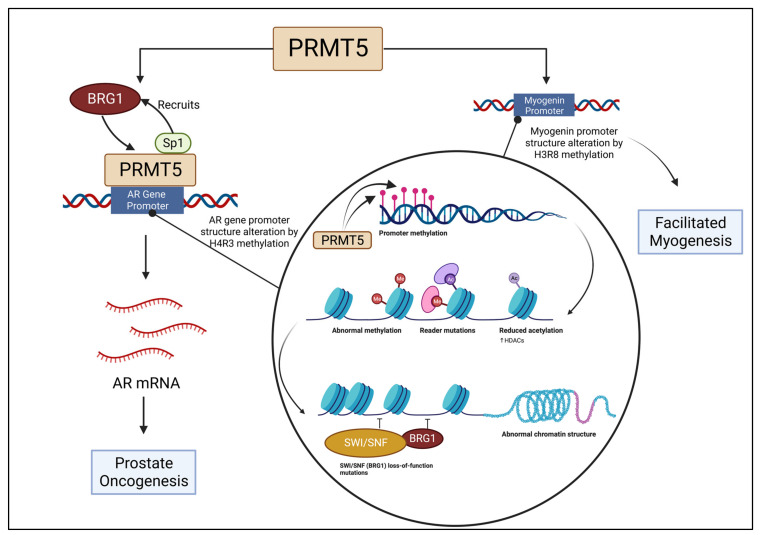
BRG1 is recruited by Sp1 to bind with PRMT5 at the auxiliary AR gene promoter region, which is altered as a result of H4R3 methylation, initiating prostate oncogenesis. The myogenin promoter is altered and structure changed by PRMT5 and BRG1 association, leading to facilitated myogenesis.

A study of prostate cancer provides evidence that PRMT5 is involved with the activation of androgen receptor (AR) transcription. PRMT5 binds to the AR gene’s proximal promoter region and is involved with symmetric dimethylation of local H4R3 within the region. A high quantity of methylated H4R3 present indicates a high expression of PRMT5 [66]. The Sp1 transcription factor for the AR interacts with PRMT5 to induce interaction between PRMT5 and the AR. In correlation with that process, a complex is formed between PRMT5 and BRG1, which is located on the AR gene’s proximal promoter region. Knockdown of Sp1 induces downregulation of both PRMT5 and BRG1 from the proximal promoter region [67]. Thus, it can be inferred from this study that the activation of AR transcription, a component of prostate oncogenesis, is reliant on a complex composed of Sp1, BRG1, and PRMT5. Figure 2 depicts the association of PRMT5 and BRG1 in modifying the AR gene promoter structure.

Certain interactions between PRMT5 and BRG1 have been theorized to be a necessary component for BRG1’s function. Without the aid of a histone-modifying enzyme, such as PRMT5, ATP-dependent chromatin remodeling enzymes, such as BRG1, will not function. Relationships between BRG1 and other proteins in the SWI/SNF complex in relation to myogenesis have been extensively studied [68]. Similarly, PRMT5 has been connected to the modification of histones, in particular H3 and H4, which impact gene regulation during the process of skeletal muscle differentiation [69]. It has been discovered that BRG1 is responsible for changing the promoter structure of myogenin in association with dimethylated H3R8 and PRMT5. Without BRG1, differentiation was downregulated. Similarly, the removal of PRMT5 also diminished differentiation levels [70]. Figure 2 depicts the association of PRMT5 and BRG1 in modifying the myogenin promoter structure.

Furthermore, overexpression of PRMT5 associated with BRG1 was found in association with an upregulation of the cell cycle regulators: Cyclin E2, Cyclin B2, and CDK4 [60]. Upregulation of these regulators has been observed in several tumor types [71,72].

PRMT5’s histone methylation function, which is linked to transcriptional activation, is found to be responsible for oncogenesis. In certain cancers, BRG1 has been observed to correspond with PRMT5 in that process. Intervention within that complex may produce favorable results in oncogenetic tumor prevention.

### 3.2. BRG1 Supresses Tumor Development and Regulates Transcription of Myc/Max/Mad Genes in Connection with PRMT5

It has been observed that downregulation of the tumor suppressor genes suppressor of tumorigenicity 7 (*ST7*) and nonmetastatic 23 (*NM23*) have been directly correlated with a downregulation of PRMT5. Such downregulation was determined to affect PRMT5-associated BGR1 complexes which performed methylation of H3R8. Direct crosstalk between BRG1 and *ST7* was discovered, but not with *NM23*. However, hBRM was discovered to be directly associated with *NM23* but not *ST7*. Interestingly, both BRG1 and hBRM interact directly with *MYT1l*, suggesting the SWI/SNF complex to be a knockout target for intellectual disability [60,73]. Furthermore, it was determined that even without overexpression of PRMT5, there is an association between BRG1 and PRMT5 [60]. Transcription levels of NM23 specifically were observed to decrease while displaying nucleoside diphosphate kinase activity, indicating high levels of metastasis within the cells overexpressing PRMT5 [74]. Within the SWI/SWF complex, BRG1 has the ability to modify the accessibility of DNase and restriction enzymes on mononucleosomes [75].

It is important to examine the complex formed between histone deacetylase 2 (HDAC2) and the mSin3A corepressor, which functions in chromatin remodeling, and its interaction with BRG1 and the SWI/SNF complex. PRMT5 is also added to the complex in the effect of notably interacting with BRG1, hBRM, and BAF45/Ini1 [76]. Histone deacetylation of K9 on H3 and H4 has been observed to enhance their methylation by PRMT5-associated SWI/SWF complexes [77]. Additionally, studies have determined that Mad-Max heterodimers suppress transcription in association with mSin3A and HDAC2 [78]. Thus, a correlation between BRG1 and this complex is of note in the chromatin remodeling process, which may play a role in Myc/Max/Mad gene repression [58].

**Figure 3:**
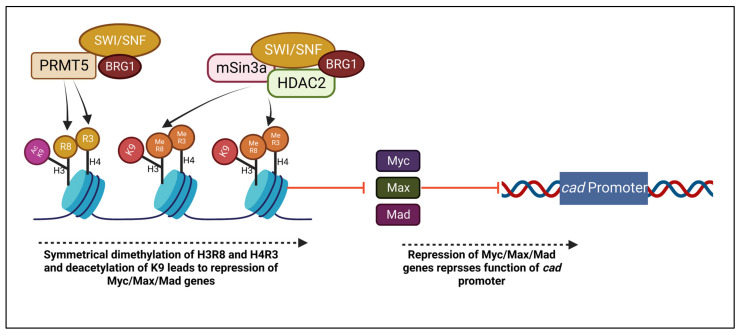
Histones H3R8 and H4R3 are symmetrically dimethylated, while K9 is deacetylated through PRMT5 and BRG1 association. A complex of PRMT5, BRG1, mSin3a, and HDAC2 repress Myc/Max/Mad genes, leading to additional suppression of *cad* promoter.

Furthermore, another study has tested a possible linkage between said transcription repression mechanism and BRG1 and hBRM with the theory that they may take part in the suppression of Myc/Max/Mad genes, which are possible targets to regulate cell proliferation [58,79]. BRG1 has also been observed to have a role in c-Myc interaction with BAF45/Ini1 and transcription activation [80]. In one study, it was hypothesized that BRG1 may repress the function of target genes, thus making the *cad* promoter region a site of interest. A knockdown of BRG1 resulted in *cad* repression which supported the hypothesis that it is a component supporting *cad* function, indicating a role of histone methylation and chromatin remodeling. Figure 3 depicts the association of PRMT5 and BRG1 in the mSin3a/HDAC2 complex. However, not all Myc/Max/Mad target genes are affected by BRG1. It was observed that *nuc* mRNA levels which were depressed by histone deacetylation through depsipeptide treatment were not even furthermore depressed within the introduction of BRG1 [58]. It can be concluded that the magnitude of repression due to BRG1 and Myc/Max/Mad interaction is dependent on the target gene.

BRG1 association with PRMT5 has been demonstrated to function as a tumor suppressor in correspondence with certain target genes. Further investigation into the binding complex and complementary nature of BRG1 and tumor suppressive complexes in the mechanisms discussed and others would be beneficial to the development of tumor regulating treatments.

## 4. BRG1 and Oncogenic RAS/RAF Pathway

Rat sarcoma virus (RAS) proteins are a family of GTPases involved in signal transduction, especially in the Ras/Raf pathway. There are three main RAS proteins, H-Ras, N-Ras, and K-Ras (referred to as KRAS in this article). KRAS is most significant to us because it is often mutated to be overexpressed in a number of cancers, leading to excessive cancer proliferation. Activating mutations are most common in pancreatic, thyroid, colorectal, and lung cancers (95–35%) [81].

Mitogen-activated protein kinases (MAPKs) are signaling pathways that regulate cellular growth, stress responses, differentiation, and viability [82,83]. Four cascades have been identified within MAPKs, including extracellular signal-regulated kinases 1 and 2 (ERK1/2), c-Jun N-terminal kinase (JNK), p38, and ERK5. ERK’s 1 and 2 (ERK1/2) are essential for regulating cell signaling and play a role in tumorigenesis [84]. ERK1/2 is stimulated in response to the sequential phosphorylation of Ras/Raf proteins, including several MAP3Ks and MAPK/ERK kinases (MEK) [85]. The Ras/Raf/MAPK/ERK1/2 pathway is one of the most prolific signal transduction pathways for tumorigenesis [84,86]. MAPK cascade signals enter the nucleus and have been proven to modulate post-transcriptional genes through interaction with transcription factors and chromatin remodeling enzymes, such as SWI/SNF [87]. Thus, an analysis of BRG1’s interaction with other proteins of the ERK1/2 pathway would be beneficial to prescribe treatment for the pathway. It is also noteworthy, considering PRMT5’s interaction with BRG1, that knockdown of PRMT5 interaction with the Ras/Raf and MEK proteins in correspondence with the ERK1/2 pathway has been exhibited to directly downregulate ERK1/2 in combination therapies [88].

### 4.1. BRG1 and Oncogenic KRAS in Cancer Formation

Previous research has discovered that BRG1 is an important protein involved in oncogenic KRAS-induced tumor growth of acinar and lung cells. However, depending on the origin of cancer development, BRG1 may act as either a suppressor or promoter.

Oncogenic KRAS is a necessary mutation for the formation of pancreatic ductal adenocarcinoma (PDAs). A total of 90% of human PDA samples have a KRAS mutation [89]. BRG1 serves a dual role in this process as a KRAS supporter and KRAS antagonist. Pancreatic ductal adenocarcinoma may be formed via PANIN, MCN, and IPMN. BRG1 has been shown to block IMPN formation and prevent oncogenic KRAS-driven PDA formation from IPMN [90]. Further study, however, found that BRG1 regulates SOX9 transcription and supports oncogenic KRAS-induced PANIN formation as well as oncogenic KRAS-induced PDA derived from PANIN [50]. Together, these studies support BRG1 as a key protein within oncogenic KRAS-initiated pancreatic cancer. Furthermore, these studies highlight the dual role of BRG1 as a promoter or suppressor of cancer, even within the same cancer of varying origin.

Research in NSCLC has supported BRG1 as an instigator of KRAS-induced cancer. KRAS mutant tumors with active BRG1 were more proliferative than KRAS mutants without BRG1 expression [43]. Another study found that BRG1 inactivation alone did not promote tumor progression. Although, the inactivation of BRG1 and P53 as well as the activation of oncogenic KRAS resulted in highly penetrant lung adenocarcinomas compared to the inactivation of just p53 and activation of oncogenic KRAS. Furthermore, when treated with an oxidative phosphorylation inhibitor, only the growth of cells with BRG1 inactivation was inhibited [91]. Therefore, BRG1 has a role in protecting KRAS cancers. 

Patients with NSCLC which present a mutation that inhibits BRG1 expression had increased mutations in the KRAS gene compared to those without BRG1 mutations [92]. Conversely, KRAS mutant NSCLC patients were more likely to have intact BRG1 than those with KRAS wildtype [43]. Furthermore, NSCLC patients with both KRAS and BRG1 mutations had worse survival outcomes in immunotherapy and non-immunotherapy treatments [93]. The increased rates of KRAS mutations in BRG1 mutant patients as well as the worse outcomes for patients with co-mutations may suggest a role for BRG1 in reducing the effects of oncogenic KRAS and cancer formation. 

Oncogenic RAS-induced senescence has been shown to regulate BRG1/BRCA1 interactions, and RAS knockdown increased BRG1 expression. Furthermore, BRG1’s association with promoters CDKN2a and CDKN1A to express p16 and p21 is upregulated during RAS knockdown-induced senescence [94].

Further BRG1/KRAS interaction in other cancers may support a greater understanding of how to properly treat KRAS mutated cancers. 

### 4.2. BRG1 and ERK/MAPK

It has been observed that BRG1 is inactivated as a result of ERK1/2 phosphorylation. However, BRG1 reactivation was observed upon the introduction of general protein phosphatase inhibitors [95]. A study examined the regulation of a complex comprising BRG1 and the heat shock factor 4b (Hsf4b) protein in response to ERK1/2 activation and inactivation [96]. Heat shock factors are generally involved with acute stress regulators through the regulation of the transcription of genes responsible for stress protein and molecular chaperone production, such as heat shock protein 70 (HSP70) [96,97]. However, alterations in the roles of heat shock factors have been observed through the malignant transformation of cells [98]. Notably, Hsf4 involves itself in olfactory neurogenesis and has been noted to be overexpressed in liver and colon cancer [99,100,101]. A previous study in which HeLa cells were co-transfected with Hsf4b as well as active MEK resulted in phosphorylation and an increase of ERK1/2 activity [102]. Experiments confirmed these findings and additionally discovered a greater association between slow-migrating BRG1 and Hsf4b. Additional experiments in HeLa cells transfected with Hsf4b and combinations of MEK and dual specificity phosphatase 26 (DUSP26) produced more significant results [96]. DUSP26 has been observed to be a tumor suppressor and an oncogene in different cellular contexts and is able to inactivate MAPK in vivo [103]. Experiments revealed that an association between BRG1 and Hsf4b is indeed upregulated by the expression of MEK. Similarly, a lack of MEK or a presence of both MEK and DUSP26 downregulated the correspondence between BRG1 and Hsf4b. It can thus be concluded that activation of MAPK and ERK1/2 promotes BRG1 and Hsf4b association. Such association was determined to be cell cycle-dependent and alters Hsf4b’s ability to bind to DNA, resulting in the negative effects noted above [96,103]. Further research into the binding of BRG1 and Hsf4b through the activated ERK1/2 pathway would be beneficial in inhibiting the complex. DUSP26 has presented itself to be a promising inhibitor, and its interactions with individual components of the ERK1/2 pathway should be studied more.

BRG1 and the ERK1/2 pathway exhibit similar notable responses to treatment. In vascular smooth muscle cells (VSMC), the introduction of BRG1 overexpression proved to increase the amount of protein expression of proliferating cell nuclear antigen (Pcna), an assisting protein to DNA polymerase in several malignant cell types, and platelet-derived growth factor (Pdgfα) [104,105]. As a result, VSMC proliferation increased. In a BRG1-shRNA group, where BRG1 expression was silenced, the amount of Pcna, Pdgfα, as well as neurotrophin-3 (Ntf3), were all reduced. Hydrosulfuric acid presented itself as an effective inhibitor of the three proteins. Phosphorylation of ERK1/2 displayed a similar trend in its activity as BRG1, Pcna, Pdgfα, and Ntf3. The results of this study indicated that hydrosulfuric acid is an effective inhibitor of VSMC proliferation through the MAPK pathway when there is a downregulation of BRG1 [106].

Studies have suggested that casein kinase 2 (CKs) is a mediator of BRG1 phosphorylation. However, the effect of CK2 phosphorylation of BRG1 is only speculatory. One study indicated that it modified the organization of telomeres as well as debilitating topologically associating domain (TAD) boundaries [107]. Another study asserted that it involves BRG1 with the nuclear structure and overall genomic organization [108]. Hyperphosphorylation of BRG1 by CK2 in mitosis is not the only source of its phosphorylation, as noted earlier in this section. MAPK also serves as a phosphorylation mediator of BRG1 in mitosis, thus acting as a controller of its chromatin remodeling function. It is inferred that the sites of phosphorylation by CK2 and MAPK are different and thus may not be the only acting kinases on BRG1 [109]. Further investigation into the existence of such other kinases and whether their association with BRG1 is similar or different would be beneficial. Additionally, besides determining if such kinases act independently, the possibility of these kinases acting in conjunction with each other should be explored.

There is significant evidence that BRG1 interacts with numerous components of the Ras/Raf/MAPK/ERK1/2 pathway. Furthermore, BRG1 has been noted to respond to treatment in concert with the ERK1/2 cascade. Activation of the ERK1/2 cascade has also presented itself to promote BRG1 interaction with other proteins. Associations between BRG1 and other proteins in correlation with cell malignancy beyond those noted in this article should be explored further.

## 5. Conclusions

In conclusion, our review emphasizes the importance of research into BRG1-related pathways in many cancers, as well as the possibility of success of BRG1 knockdown in conjunction with another treatment. Much of the evidence suggests BRG1 supports autophagy and inhibits apoptosis. These are two methods by which BRG1 supports cancer growth and prevents treatment. BRG1 works with cancer-specific proteins to inhibit apoptosis, and the knockdown of BRG1 may support other treatments such as chemotherapy. PRMT5 has demonstrated itself to both bind and associate with BRG1 to accomplish histone methylation. Through such methods, BRG1 is able to function within complexes to regulate both gene activation and repression. Notably, several of those complexes regulate tumor suppressor genes. Research into protein binding within such complexes may be useful to specify targets for treatments to either enhance or diminish BRG1’s association depending on the target gene as a preventative method. BRG1 has displayed a role in oncogenic KRAS-induced cancers with directionality seemingly dependent on cancer type. BRG1 associates with several proteins within the Ras/Raf/MAPK/ERK1/2 pathway and is promoted to form complexes with other proteins when ERK1/2 is activated. Given that BRG1 and PRMT5 both play a similar role in apoptosis and autophagy and are both involved in the RAS/RAF pathway, further insight into the effects of dual knockdown may show promising results. This paper hopes to emphasize the possibility of cancer treatments benefiting from the co-treatment of BRG1.

## Data Availability

Not applicable.
